# Glances at the Hospitals

**Published:** 1901-02-16

**Authors:** 


### GLANCES AT THE HOSPITALS.

### THE CLAYTON HOSPITAL, WAKEFIELD.

Exclusive of legacies the income for the year 1899 amounted to £3,364, and the expenditure to £2,858. The annual subscriptions were £492, showing a falling off of £33, when compared with the previous year, but the workpeople's own contributions were £1,008, against £846 in 1898. A children's ward is being built, and the expense of it is being defrayed by Mrs. Milnes-Gaskell. It will accommodate 28 children's cots, and Mrs. Beardsley has placed £400 in the hands of the committee to provide new consulting-rooms. The hospital seems to be flourishing. At the beginning of the year there were 49 patients in the hospital; 519 were admitted during the year, making a total of 568 under treatment. Of these 430 were discharged cured, 48 relieved, 5 not relieved, 33 died, and 52 remained under treatment. It should be noted that 12 of the deaths occurred within 48 hours of admission. Altogether 3,728 out-patients were treated, and 2,696 were discharged cured, 486 relieved, 34 for other reasons, 29 died, and 484 remained on the books. A statistical report of the in-patients is given. It shows the numbers suffering from the various diseases and also numbers cured, relieved, not relieved and died. Five cases of fracture of base of the skull are recorded, and two of these recovered. 169 operations were performed but no results are given, a fault which we hope will be remedied in the report for 1900. A statement of anæsthetics used should also be printed, and a table showing the cost per head and the cost per bed occupied.

### THE NATIONAL SANATORIUM FOR CONSUMPTION AT BOURNEMOUTH.

In looking over the hospital reports one is often tempted to say that they are unnecessarily long and are weighted with lists of names of past and present donors; but the report of the National Sanatorium is almost too short. Nevertheless it conveys much information. The committee say that in spite of every endeavour in economy, the ordinary expenditure is higher than it was in 1898; and that unless there be an increase in subscriptions and donations there will be a deficit of £200 at the end of 1900. It is the duty of the charitable people throughout England to see that this shall not occur. So much for the ordinary expenditure; but when we turn to the extraordinary expenditure things are rather worse, for of £1,000 spent on sanitary arrangements, only £345 has been received. That these improvements were most desirable we have no doubt; but we are not much in favour of embarking on any building operations unless the money be actually in hand, or that there is a certainty in the immediate future of obtaining it. Mr. G. J. Fenwick gave £1,000 for the purpose of setting free from weekly payments for twelve weeks annually for a period of fifty years, two patients who have received nomination; and an anonymous donation of £100 has been received for the erection of open-air shelters. There were 59 patients in residence at the beginning of the year, and 200 admissions during it, making a total of 259. Of these 101 were much improved, 73 were improved, 29 not improved, 2 were transferred, 3 died, and 51 were under treatment. The average stay of each patient was 62 days, and the total cost of each patient was £1 3s. 11d. per week.

